# Assessment of the quality of cleaning of surfaces in care rooms of intensive care units: feasibility of the use of ATP-metry

**DOI:** 10.1186/2047-2994-4-S1-P29

**Published:** 2015-06-16

**Authors:** J Stuck, P Batailler, P Saviuc, B Allenet, M-R Mallaret

**Affiliations:** 1 Pharmacy, University Hospital, Grenoble, France; 2 Hygiene Unit, University Hospital, Grenoble, France; 3 Univ. Grenoble-Alpes, TIMC-IMAG (UMR CNRS 5525), CHU de Grenoble, Pôle Pharmacie , France; 4 Univ. Grenoble-Alpes, TIMC-IMAG (UMR CNRS 5525), CHU de Grenoble, Pôle Santé Publique, Grenoble, France

## Introduction

Adenosine triphosphate (ATP) is the basic energy molecule for all living cells. Its presence on a surface is a sign of contamination by microorganisms. ATP-metry is an ATP detection technique based on chemiluminescence. It is used in the food industry to assess the quality of cleaning of surfaces.

## Objectives

To assess the value of using ATP-metry to control the quality of cleaning of care rooms (CR) surfaces in the intensive care units of a University Hospital.

## Methods

A prospective study comparing microbiological sampling of surfaces made with contact agar plates and ATP-metry of swabs throughout the intensive care units. Seven sampling points were defined in each CR (sink, middle and edge of the workbench, bottles, drug storage shelf, refrigerator, garbage collector). The criteria were the number of bacteria Colony-Forming Units (CFU) per 25 cm^2^ agar and the number of Relative Light Units (RLU) per 100 cm² swabbed. The data were processed using Excel and a Spearman correlation (r_S_) was used (StatView).

## Results

Eighty six points were sampled in 13 CR. The environment was considered well controlled in 7 CR, but cleaning needed to be improved in the others (especially drug storage shelves and sinks). Significant poor correlation (r_S_ = 0.25; p = 0.02) was found between the number of CFU (median 2, range 0-300) and the number of RLU (median 70, range 8-619). The results were the same when correlating point by point.

## Conclusion

In this study, the presence of ATP was not correlated to microbiological contamination. This finding may be explained by different hypotheses: the amount of ATP may be underestimated due to its intracellular localization or overestimated in case of contamination of the environment by ATP of non-microbiological origin. ATP-metry does not seem to be a technique of choice for monitoring the quality of surface cleaning in the care rooms of intensive care units.

## Disclosure of interest

None declared.

